# CMap analysis identifies Atractyloside as a potential drug candidate for type 2 diabetes based on integration of metabolomics and transcriptomics

**DOI:** 10.1111/jcmm.15357

**Published:** 2020-05-29

**Authors:** Hailong Li, Xiaodong Shi, Hua Jiang, Junren Kang, Miao Yu, Qifei Li, Kang Yu, Zhengju Chen, Hui Pan, Wei Chen

**Affiliations:** ^1^ Department of Clinical Nutrition Peking Union Medical College Hospital Chinese Academy of Medical Sciences and Peking Union Medical College Beijing China; ^2^ Department of Health Medicine Peking Union Medical College Hospital Chinese Academy of Medical Sciences and Peking Union Medical College Beijing China; ^3^ Institute for Emergency and Disaster Medicine Sichuan Provincial People's Hospital Sichuan Academy of Medical Sciences School of Medicine University of Electronic Science and Technology of China Chengdu China; ^4^ Department of Endocrinology Peking Union Medical College Hospital Chinese Academy of Medical Sciences and Peking Union Medical College Beijing China; ^5^ Pooling Medical Research Institutes Hangzhou China; ^6^ Beijing Key Laboratory of the Innovative Development of Functional Staple and the Nutritional Intervention for Chronic Disease Beijing China

**Keywords:** Atractyloside, gene, metabolomics, pathway, type 2 diabetes

## Abstract

**Background:**

This research aimed at exploring the mechanisms of alterations of metabolites and pathways in T2D from the perspective of metabolomics and transcriptomics, as well as uncovering novel drug candidate for T2D treatment.

**Methods:**

Metabolites in human plasma from 42 T2D patients and 45 non‐diabetic volunteers were detected by liquid chromatography‐mass spectrometer (LC‐MS). Microarray dataset of the transcriptome was obtained from Gene Expression Omnibus (GEO) database. Kyoto Encyclopedia of Genes and Genomes (KEGG) database was used to conduct pathway enrichment analysis. Connectivity Map (CMap) was employed to select potential drugs for T2D therapy. In vivo assay was performed to verify above findings. The protein expression levels of *ME1*, *ME2* and *MDH1* were detected by Western blot to determine the status of NAD/NADH cofactor system.

**Results:**

In our study, differentially expressed metabolites were selected out between healthy samples and T2D samples with selection criteria *P* value < .05, |Fold Change| > 2, including N‐acetylglutamate and Malate. Genes set enrichment analysis (GSEA) revealed that 34 pathways were significantly enriched in T2D. Based on CMap analysis and animal experiments, Atractyloside was identified as a potential novel drug for T2D treatment via targeting *ME1*, *ME2* and *MDH1* and regulating the NAD/NADH cofactor system.

**Conclusion:**

The present research revealed differentially expressed metabolites and genes, as well as significantly altered pathways in T2D via an integration of metabolomics, transcriptomics and CMap analysis. It was also demonstrated that comprehensive analysis based on metabolomics and transcriptomics was an effective approach for identification and verification of metabolic biomarkers and alternated pathways.

## 1 INTRODUCTION

Type 2 diabetes (T2D) has become one of the most challenging diseases for its alarmingly high rate rise in modern society. Many factors are associated with T2D development and progression, among which excessive intake of sugar or lipid and genetic factors might greatly contribute to the occurrence of T2D.^1^ Previous researches proved that insulin was the regulator of glycogen metabolism and absorption, which was crucial for the energy and body balance.^2^ To further understand the efficacy of insulin in T2D treatment, this research focused on whether and how insulin could influence glucose absorption. Insulin resistance is a common risk factor in T2D and cardiovascular disease progression.^3^ Former studies revealed that the activation impairment of insulin was typically happened in T2D patients, which could be attributed to a polygenic disorder.^4^

From the perspective of metabolomics, T2D is associated with metabolism of lipid and glucose, and previous study elucidated the defects in glycogen synthesis and transportation which may lead to decreasing glucose uptake in T2D patients.^5^ In recent years, metabolomic approaches have been conducted in T2D which successfully acquired mechanism insights and found potential metabolic biomarkers including amino acids, cycle intermediates, fatty acids.^6^ Li‐Gao et al^7^ defined T2D patients and healthy people with post‐prandial metabolite profiles, which had a satisfactory stratification performances compared with medical methods. Despite the alteration of glycogen, many metabolites demonstrated differences between samples from healthy people and T2D patients, and Padberg et al^8^ uncovered a metabolic signature composed of increasing glyoxylate, which was a potential novel biomarker for the detection of T2D in early stage. With the support of Metabolomics Standard Initiative (MSI), experiments in vivo demonstrated urinary changes in T2D and revealed an altered urinary metabolism.^9^

Genetic signatures were inheritable and complex which were hard to capture. Previous studies performed with transcriptomic profiling indicated that in T2D myocytes and myoblasts cells, the mitochondrial regulatory genes were dysregulated in a moderate way, suggesting that the alterations might occur at post‐transcriptional level.^3^ Hansen et al^10^ revealed an exercise‐induced regulation by amino acids biosynthesis and metabolism related genes (*GLDC*, *NOSI* and *PSPH*), responding to the consistently lower level of arginine, cysteine and glycine in plasma.

Combination of metabolomics and transcriptomics, as an emerging method, has been applied in many fields. Transcriptomics could provide a deeper insight into the mechanisms of diseases from a transcriptional and post‐transcriptional level. Through integration with transcriptomic data, the results from metabolomic analysis would be more comprehensive and valid. In diabete fields, studies performed with the integration of metabolomics and transcriptomics were still in initial stage and remained limited. An application of integrated methods was used in studies in vivo. Dumas *et al* highlighted the importance of multilevel datasets in the establishment and understanding of genes and metabolites, and the analysis of T2D rat congenic series based on gene‐metabolites network characterized the role of certain genes.^11^ Solimena et al^12^ found signature genes included TMEM37 that repressed Ca^2+^ influx in beta cells, and transcriptomic changes in down‐regulation of signatures in islets were captured.

Atractyloside (ATR), a diterpenoid glycoside, has been found in many East Asian plants (Asteraceae, Atractylis) and used as medical herbs in traditional Chinese medicine (TCM).^13^ Lots of studies revealed the biochemistry and toxicity of ATR, which made it convinced for disease treatment. For example, ATR was shown to exert a diuretic effect on oedema and be liver protective according to pharmacological clinical researches on humans.^14^ More importantly, Shan et al^15^ identified it as a mild hypoglycaemic agent in splenocytes, suggesting its potential therapeutic effects for diabetes. However, precise molecular mechanism of ATR in T2D remains unclear.

Our study intended to explore more effective biomarkers and potential drugs for T2D diagnosis and treatment in the future. With the application of metabolomic methods, we detected the level of metabolites and revealed enrichment pathways. By using transcriptomic data, gene expression information was taken into account. Based on CMap and animal experiments in vivo, we intended to figure out significant signatures which could be function as potential medicine for the treatment of T2D.

## 2 MATERIALS AND METHODS

### 2.1 Sample collection

Human plasma samples were collected from 45 healthy patients and 42 T2D patients from Peking Union Medical College Hospital. Before the study was conducted, all participants had signed informed consents. The criteria of the T2D patients to this study were fasting blood glucose (FPG) ≥ 7 mmol/L, HbA1c < 8.5%. The healthy patients were aged 22‐55 years and weighted 48‐107 kg with a body mass index (BMI) of 18‐30 kg/m^2^, free from any major disease or pregnancy. The T2D patients were aged 34‐67 years and weighted 65‐138 kg with a BMI of 25‐40 kg/m^2^, treated with most one oral anti‐diabetic drug. T2D patients agreed to stop treatment with oral anti‐diabetic agents during the study. Patients went through a washout period of 4 week before sample collection and abstained from alcohol during the study; diet was controlled throughout the study. This study was authorized by the ethic committee of Peking Union Medical College Hospital.

### 2.2 Sample treatment

Blood samples in our study were extracted from a peripheral vein. Collected blood samples were preserved in ice and then centrifuged for 15 minutes at 1500 *g* at 4°C. The plasma supernatant was stored at −80°C for future analysis. About 40 μL sample was dissolved at −20°C in 1 mL acetonitrile/isopropanol/water (3:3:2) solvent. The supernatant containing metabolites was dried in a vacuum centrifuge. Subsequently, all of the samples were mixed with 30 μL acetonitrile/water (1:1) solution and went through 0.22 μm PVDF filters (Phenomenex), 2 μL trimethylsilyl propionic acid. After those treatments, they were prepared and used for later analysis.

### 2.3 Liquid Chromatography‐Mass spectrometry (LC‐MS) analysis

LC‐MS analysis was conducted by an UHPLC system (Infinity 1290; Agilent Technologies). The system was connected to a Q‐TOF/MS instrument (Agilent 6410 Q‐TOF MS/MS, Agilent Technologies). LC‐MS data were collected and analysed by MassHunter B.03.01 (Agilent Technologies). The injection of each sample was performed twice, 2 μL for negative ionization mode analysis and positive ionization mode analysis, respectively. The column compartment temperature was 40°C, the setup of flow rate was 0.4 mL/min, the total separation time of two ionization modes was 20 minutes and the autosampler temperature was kept at 4°C. The mobile phase comprised Solvents A (5 mmol/L 0.1% formic acid) and Solvents B (acetonitrile with 0.2% acetic acid). By spiking the pooled serum samples containing mixtures of five standard metabolites, the identities of metabolites were proved.

### 2.4 Integration of metabolomic data and transcriptomic data

The metabolic profiling was integrated with transcriptomic data to unveil differentially expressed signalling pathways between T2D group and non‐diabetic group. Transcriptomic data GSE25724 from Gene Expression Omnibus (GEO) with corresponding platform GPL96 were employed for sifting out differentially expressed genes (DEG) between T2D and non‐diabetic samples. Microarray analysis was performed to evaluate differences in the transcriptome of T2D human islets compared with non‐diabetic islet samples. Specifically, human islets were isolated from pancreas of 7 non‐diabetic (age 58 ± 17 year; gender, 4 males/3 females; body mass index, 24.8 ± 2.5 kg/m^2^) and 6 type 2 diabetic (age 71 ± 9 year; gender, 3 males/3 females; body mass index, 26.0 ± 2.2 kg/m^2^) organ donors by collagenase digestion followed by density gradient purification. Thereafter, human islets were hand‐picked and cultured 2 days in M199 culture medium. DEGs and metabolites were selected out via R studio (https://www.rstudio.com/), which were shown in both volcano plots and heat maps. Thereafter, the metabolites were converted to KEGG (Kyoto Encyclopedia of Genes and Genomes) Database (http://www.genome.jp/kegg/kegg1.html). Orthogonal Partial Least Squares Discriminant Analysis (O‐PLSDA) and ROC analysis between two groups were conducted using Metaboanalyst 3.0 (http://www.metaboanalyst.ca). Based on Gene Set Enrichment Analysis (GSEA) (http://software.broadinstitute.org/gsea/), significantly enriched signalling pathways were determined. Protein‐protein interaction (PPI) network was constructed via String (https://string‐db.org/) to further investigate the interaction between related genes and KEGG pathways. Meanwhile, the interaction network between DEGs was established through Cytoscape (http://www.cytoscape.org). Venny diagram was plotted on Venny 2.1.0 (http://bioinfogp.cnb.csic.es/tools/venny/). Connectivity map analysis was conducted referring to the protocols by Li et al and Kappler et al Based on connectivity map analysis, ATR was selected out for further analysis.

### 2.5 Animals maintenance and in vivo treatment

Five male C57BL/6J mice (4 weeks old) as control group and ten male db/db diabetic mice (4 weeks old) were all purchased from NanJing Better Biotechnology Co., Ltd. The db/db diabetic mice were randomly divided into two groups, and one of which was treated with 30 mg/kg/d ATR for 12 weeks during the experimental progresses. The other one group of db/db diabetic mice and control group were treated with water. Mice were housed in a suitable environment (23 ± 2°C and 70% humidity) with a 12‐hour light‐dark cycle and had free access to water and standard chow food for 6 weeks. All studies were in accordance with the Guide for the Care and Use of Laboratory Animals (National Institutes of Health, USA) and approved by the Animal Care and Ethics Committee.

### 2.6 Dose fixation via in vivo assay

To determine doses of Atractyloside (ATR) used in the following experiments, T2D mice were treated with a series of ATR (Sigma Aldrich) concentration (5, 10, 15, 20, 25, 30, 35 and 40 mg/kg) by intragastric administration. Finally, 30 mg/kg ATR was found to be most effective based on inspection of fasting blood glucose.

### 2.7 Measurements of physiological and serum chemical parameters in blood

Measurements were made after 12 hours of fasting. Food intake level, bodyweight and blood glucose were measured weekly. At the end of experiment, blood samples were collected from the tail vein. FBG was evaluated by Glucometer (Arkray). A commercial kit obtained from Jiancheng Biology Engineering Institute (Nanjing, China) was utilized to determine total cholesterol (TC) and total triglycerides (TG). HbA1c and insulin were measured by ELISA kits (Morinaga Institute of Biological Science, Tokyo, Japan). The content of malate was determined by LC (Agilent Technologies).

### 2.8 Glucose tolerance test

Mice were fasted for 12 hours and then intraperitoneally injected glucose at the dosage 2 g/kg bodyweight. After this, blood glucose was measured at 15, 30, 60, 90 and 120 minutes after injection.

### 2.9 Western blotting

Western blotting was performed as follows. First, extraction Kit (Epigentek) was utilized to isolate protein samples. Thereafter, 8% SDS‐PAGE gel was employed to separate proteins, which were then transferred onto PVDF membranes (Millipore). Subsequently, the membranes were blocked with phosphate‐buffered saline with Tween‐20 (PBST) solution (Sigma‐Aldrich) and 5% bovine serum albumin (Sigma‑Aldrich; Merck KGaA) for 1 hour at room temperature. Subsequently, PVDF membranes were incubated with the primary antibody at 4°C overnight, including anti‐ME1 (1:1000, ab97445, Abcam), anti‐ME2 (1:2000, ab139686, Abcam), and anti‐MDH1 (1:1000, ab181091, Abcam), with β‐actin (1:200, ab115777, Abcam) being as the loading control. HRP‐conjugated anti‐rabbit antibody (GE Healthcare) was used as secondary antibody. The protein bands were visualized by LAS‐300 imaging system (Fujifilm).

### 2.10 Statistical analysis

Data were expressed as mean ± SD. GraphPad Prism 6 software was used to perform statistical analysis. Multivariate data are expressed as mean ± SD and evaluated by analysis of variance (ANOVA), Tukey's test was performed in all pairwise comparisons. Statistical significance was set at a level of *P* < .05.

## 3 RESULTS

### 3.1 Metabolites profiling of T2D showed significant difference compared with normal

**Figure 1:**
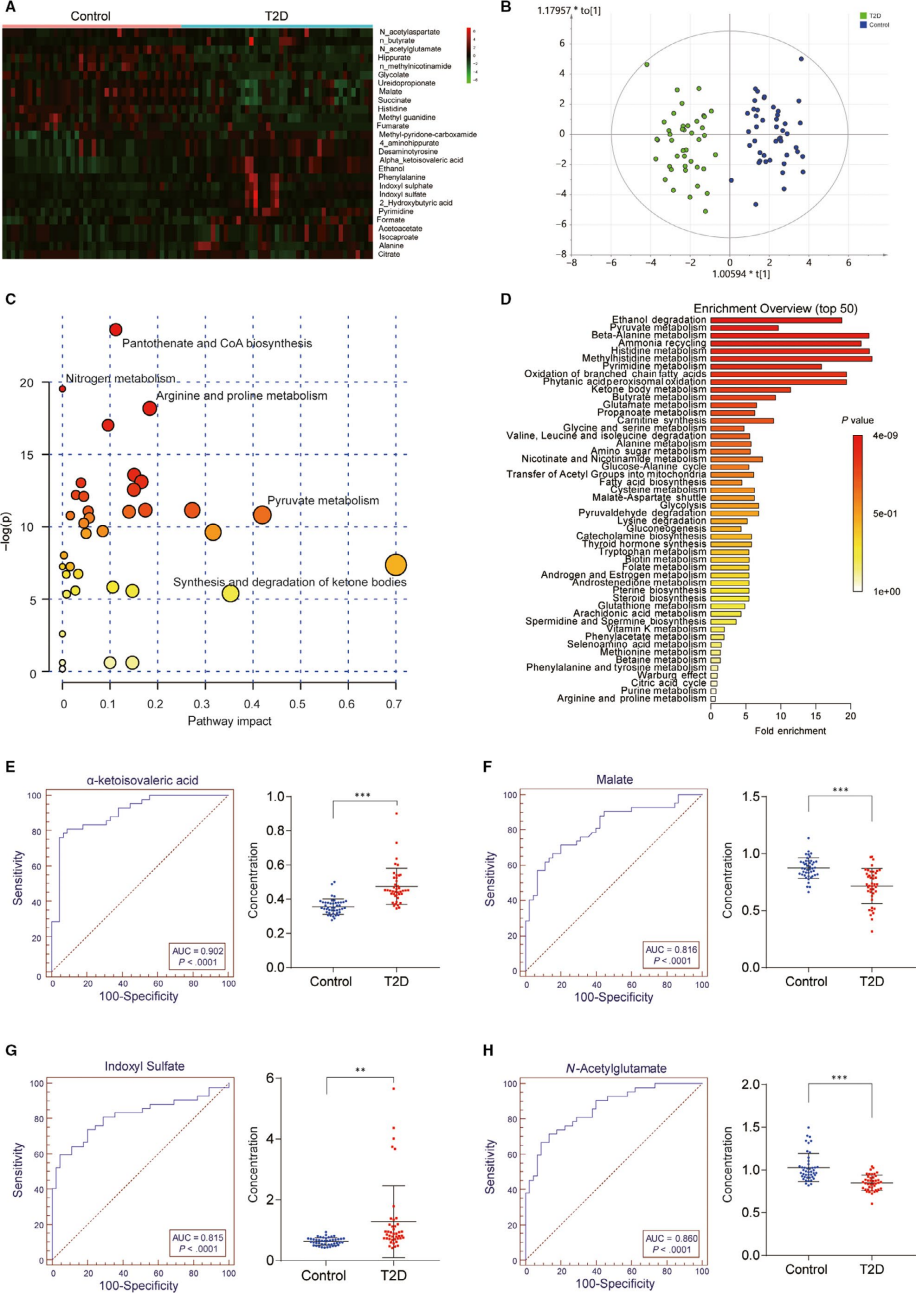
Metabolomic analysis results were significantly different between healthy group and T2D patients and the ROC curves of the potential biomarkers for T2D. A, Heat map of the differentially expressed metabolites between healthy volunteers and T2D patients. Eleven metabolites were down‐regulated in T2D patients compared with the healthy patients and represented by green, while 16 metabolites were overexpressed and indicated by red; B, O‐PLSDA score plot for the samples remarkably separated the T2D samples from the controls samples. C and D, Pathway enrichment analysis showed the top 50 pathways that were most significantly altered in the T2D. Compared with the control group, *P* < .05. E, α‐ketoisovaleric acid showed an area under curves (AUC) of 0.902 (*P* < .0001). F, Malate showed an AUC of 0.816 (*P* < .0001). G, Indoxyl sulfate showed an AUC of 0.815 (*P* < .0001). H, N‐Acetylgutamate showed an AUC of 0.860 (*P* < .0001). **P < .01; ***P < .001, compared with the control group

**Figure 2:**
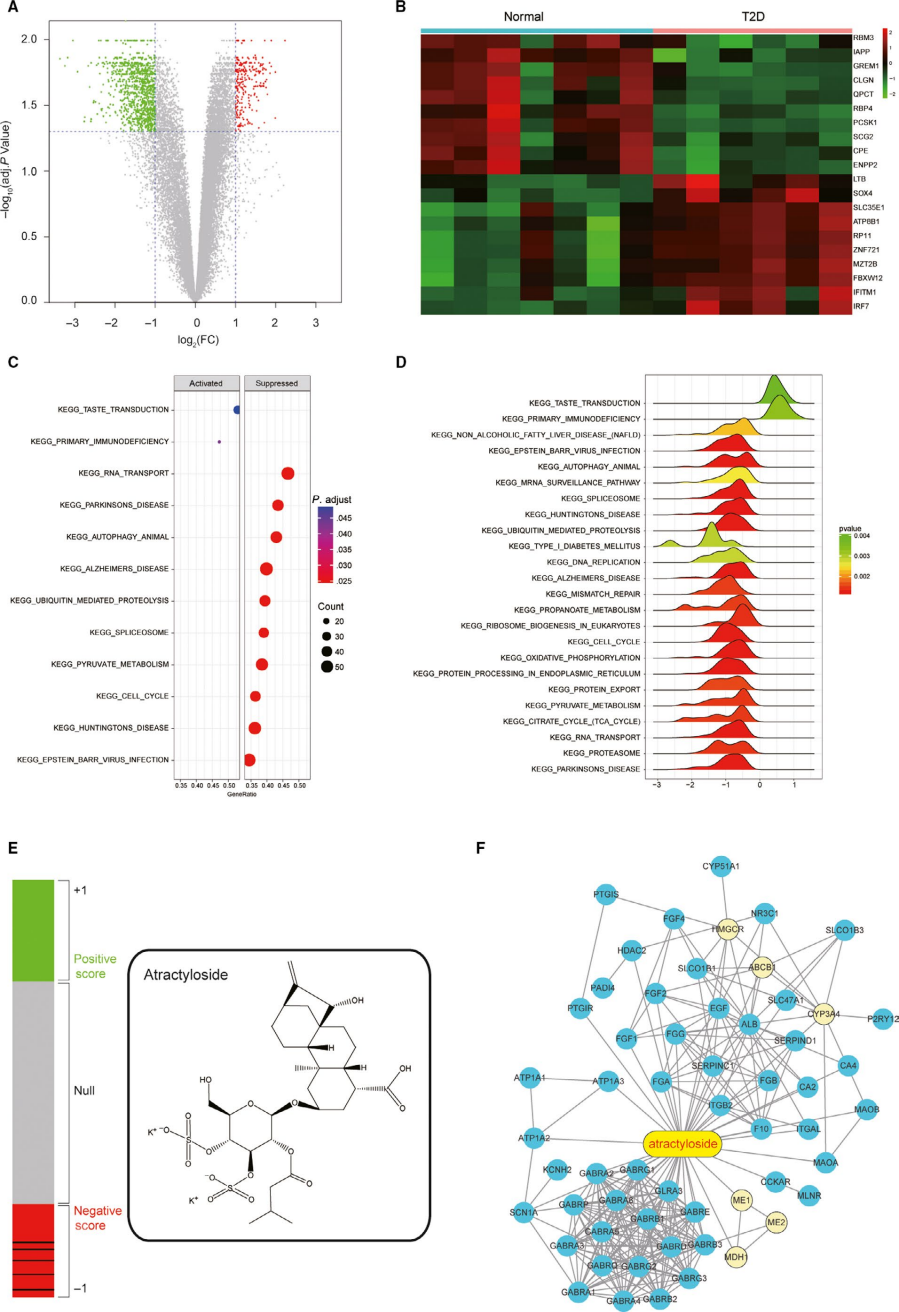
Transcriptomic analysis results indicated differentially expressed genes and pathways between healthy volunteer and T2D patients, and the CMap analysis results indicated that Atractyloside might have the treatment effect to T2D. A, Volcano plot indicated that 774 differentially expressed genes (DEGs) were screened in T2D patients in the accordance with Gene Expression Omnibus (GEO) database. Threshold was set to be log_2_|FC| > 1 and *P* < .05. B, Heat map revealed the differences of gene expression between T2D patients (Case) and the healthy volunteers (Normal). C, The dotplot illustrated the top 12 altered pathways that could lead to metabolic disorders or the occurrence of T2D. D, The joyplot indicated the top 24 altered pathways, and thereinto 2 were activated in T2D while the rest were suppressed. E, The left bar graphs reveal the Connectivity Score data of Atractyloside in the GSE25724. Lines in each colour sections represent each instance performed with the respective compound. The rounded rectangle structure of Atractyloside was illustrated in the right. F, A network of potential genes which atractyloside might target to. Six genes in yellow circle were the differentially expressed genes screened from transcriptomic data, including malic enzyme 1 (ME1), malic enzyme 2 (ME2), malate dehydrogenase 1 (MDH1), 3‐hydroxy‐3‐methylglutaryl‐CoA reductase (HMGCR), ATP binding cassette subfamily B member 1 (ABCB1) and cytochrome P450 family 3 subfamily A member 4 (CYP3A4)

A total of 87 samples consisting of 42 samples from T2D patients and 45 samples from non‐diabetes healthy volunteers were taken into account. The untargeted metabolomics analysis was performed by LC‐MS, and the results indicated that 87 valid samples were identified and measured. In addition, 27 metabolites were defined as differentially expressed in T2D patients compared with control group (*P* < .05, Figure 1A), in which 16 metabolites were up‐regulated, including ethanol, pyruvate and α‐ketoisovaleric acid, and 11 metabolites were greatly down‐regulated, including N‐acetylglutamate, succinate and malate (Table S1). Orthogonal Partial Least Squares Discriminant Analysis, a multivariate method, was adopted to analyse metabolites. As shown in Figure 1B, the O‐PLSDA score plots clearly differentiated between T2D group and control group, indicating that it was available to select metabolic biomarkers. Pathway enrichment analysis was conducted to further investigate the alteration of pathways in T2D. Top 50 metabolic pathways were found to be significantly altered (*P* < .05) in Figure 1C,D. We further explored potential diagnostic biomarkers for T2D. The diagnostic performances of these metabolites were tested and the area under curves (AUC) of α‐ketoisovaleric acid, malate, indoxyl sulfate and N‐acetylglutamate showed significant prediction of T2D (AUC > 0.8, *P* < .001) (Figures 1E and 2H). Based on metabolites profiling and model evaluation, we concluded that α‐ketoisovaleric acid, malate, indoxyl sulfate and N‐acetylglutamate might have be useful for disease diagnosis, treatment and prognosis.

### 3.2 Differentially expressed genes and pathways between T2D samples and healthy samples were determined by transcriptomic analysis

Transcriptomic data were downloaded from Gene Expression Omnibus Series GSE25724 with the corresponding GPL96 platform. A total of 774 genes were selected out as DEGs in T2D patients (Figure 2A), and the heat map revealed a significant change in gene expression between healthy people and T2D patients (Figure 2B). Both the dotplot and joyplot showed altered pathways, which may lead to metabolic disorders and even the existence of T2D (Figure 2C,D). Our study also supported the viewpoint that a broad range of genetic variation impacted T2D risk through a limited number of biological pathways.

### 3.3 Connectivity map (CMap) analysis identified ATR as a potential drug for T2D treatment

CMap is a widespread bioinformatics method to build functional connections among genes, drugs and diseases. After the ranking of CMap analysis (Table S2), compounds with positive correlation scores, null and negative scores were plotted in the bar graph, and the structure of ATR was also illustrated in Figure 2E. According to these results, a network‐based approach focused on bringing sources of information together about gene function that are most relevant to ATR was developed (Figure 2F). Six possible candidate genes that could serve as potential biomarkers for T2D diagnosis were selected, including malic enzyme 1(*ME1*), malic enzyme 2 (*ME2*), malate dehydrogenase 1 (*MDH1*), 3‐hydroxy‐3‐methylglutaryl‐CoA reductase (*HMGCR*), ATP binding cassette subfamily B member 1(*ABCB1*) and cytochrome P450 family 3 subfamily A member 4(*CYP3A4*).

### 3.4 Content of T2D‐related chemicals was significantly up‐regulated in db/db mice, which could be partially reversed with ATR treatment

**Figure 3:**
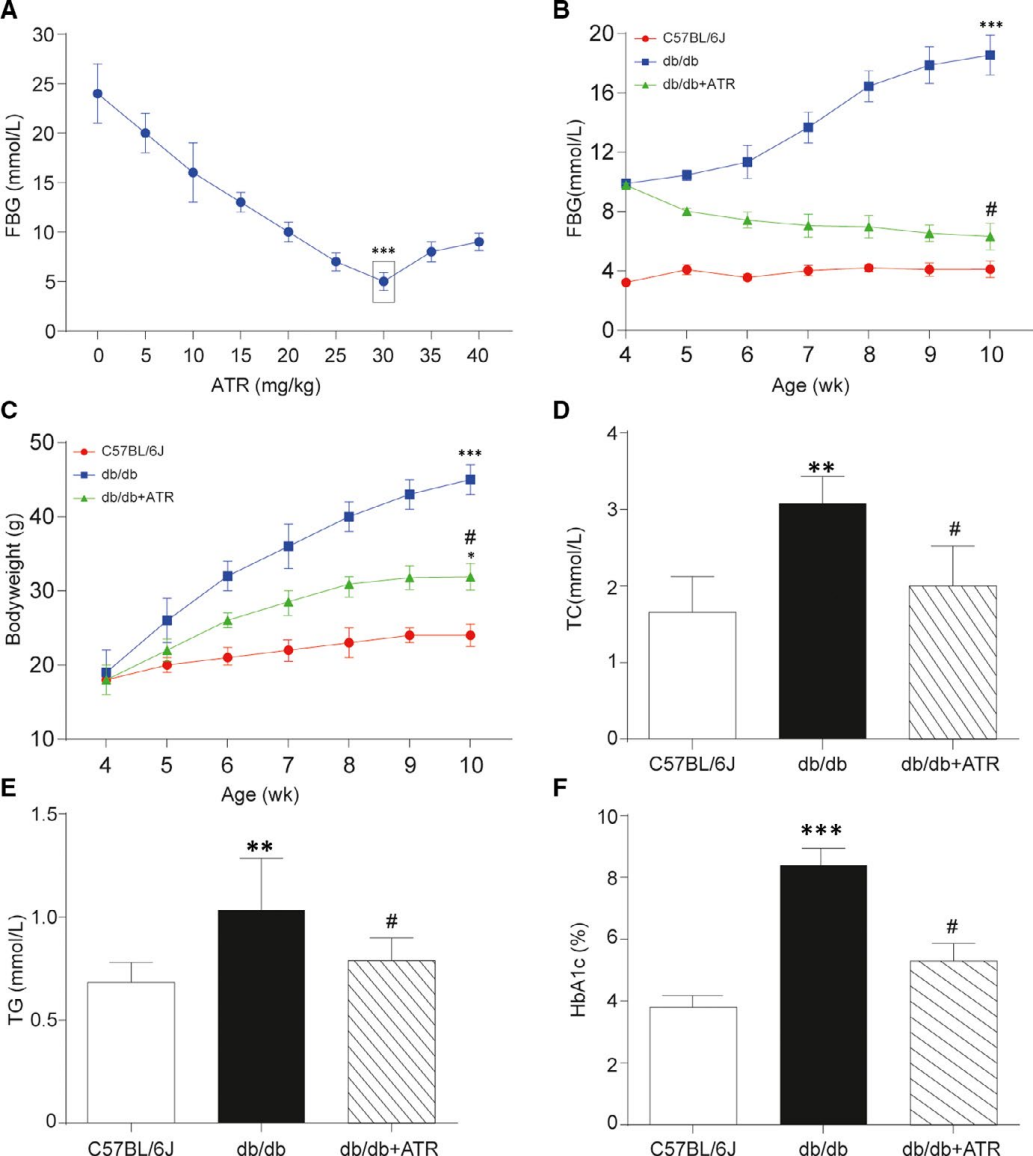
Measurement of bodyweight and serum biochemical parameters in each mice group. A, The change curve of fasting blood glucose (FBG) went along with different doses of Atractyloside. With 30 mg/kg ATR treatment, FBG got the lowest in T2D group. B, Glucose tolerance test of each group. FBG of db/db mice treated with additional 30 mg/kg ATR was significantly decreased compared with those without treatment in each week, while FBG of C57BL/6J mice remained in a low level at around 4 mmol/L. C, Bodyweight of mice in each group. The increasing of bodyweight in db/db mice compared with C57BL/6J group was suppressed with additional ATR treatment. D, Total cholesterol (TC) level of mice in each group. TC in db/db mice was much higher than that in C57BL/6J group, which was relieved in db/db mice treated with ATR. E, Triglyceride (TG) level of mice in each group. TC in db/db mice was increased compared with C57BL/6J group, which was relieved with additional ATR treatment. F, Plasma glycated haemoglobin (HbA1c) of mice in each group. The level of HbA1c was remarkably increased in db/db mice compared with C57BL/6J mice and got repressed after ATR treatment. Data = mean ±SD, n = 5 in each group, *P < .05, **P < .01, ***P < .001, compared with NC (C57BL/6J) group. ^#^ *P* < .05, ^##^ *P* < .01, compared with db/db group

**Figure 4:**
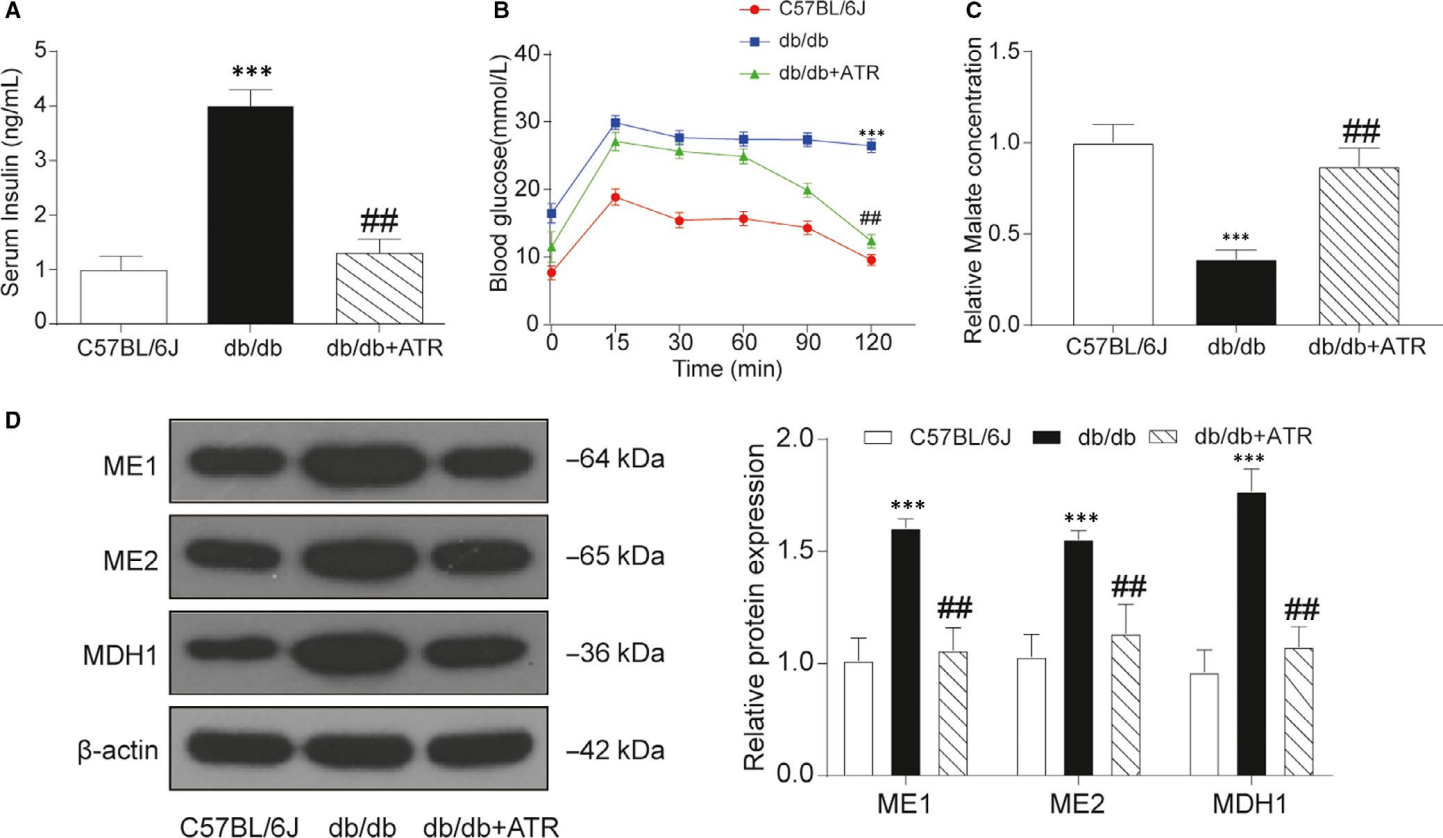
ATR partially decreased the incidences of T2D in db/db mice. A, Serum insulin level of db/db mice was notably higher than other mice. B, Results of blood glucose tested after injection of 2 g/kg bodyweight during 2 h in glucose tolerance test. The increasing of blood glucose level in db/db mice was relieved by ATR administration. C, Malate concentration detected by LC‐MS. Malate concentration was down‐regulated in T2D mice and got retrieved to baseline level with ATR treatment. D, The protein expression level of *ME1, ME2* and *MDH1* in C57BL/6J mice, db/db mice and db/db mice treated with ATR. Relative protein expression levels of ME1, ME2 and MDH1 were significantly down‐regulated in db/db mice compared with the control groups, which was partially rescued to normal expression level with ATR treatment. Data = mean ±SD, n = 5 in each group, *P < .05, **P < .01, ***P < .001, compared with NC (C57BL/6J) group. ^#^ *P* < .05, ^##^ *P* < .01, compared with db/db group

To guarantee the safety and efficiency in animal experiments, preliminary experiments were carried out with various concentrations of ATR. As shown in Figure 3A, FBG was the lowest with treatment of 30 mg/kg ATR in T2D group. Hence, 30 mg/kg ATR at a concentration of 30 mg/kg was used for further analysis in this study. Besides, some crucial physiological indexes were investigated via in vivo assay for better examination of therapeutic effects of ATR on T2D. In db/db mice, food intake was remarkably increased compared with C57BL/6J mice, but not significantly different from the untreated group (Table S3). FBG was significantly increased, which reached the level of diabetes after 10 weeks (Figure 3B). Besides, bodyweight of db/db mice was significantly heavier than that of C57BL/6J mice (Figure 3C), with TC (Figure 3D) and TG (Figure 3E) showing similar tendency. In addition, the level of HbA1c was also greatly increased in db/db mice compared with C57BL/6J mice (Figure 3F). Importantly, serum insulin level was much higher than before (Figure 4A) and the glucose tolerance test indicated that blood glucose level remained above 10 mmol/L after glucose injection in db/db mice, while with additional ATR treatment serum insulin level and blood glucose level in db/db mice were both decreased nearly to the normal level (Figure 4B). Results of LC‐MS indicated that malate concentration was down‐regulated in T2D mice, which was retrieved to baseline level with treatment of ATR (Figure 4C). As shown in Figure 4D, relative protein expression levels of NAD/NADH cofactors *ME1*, *ME2*, *MDH1* were significantly up‐regulated in T2D group compared with the control groups. In addition, the up‐regulation of *ME1*, *ME2*, *MDH1* in T2D group could be partially rescued to normal expression level with treatment of ATR. Despite ATR could increase their protein level, the accurate mechanism of action remained unclear.

Based on these data, ATR could partially restored the ability of glucose regulation by alleviating the increase of bodyweight, FBG, TG, TC, HbA1c and serum insulin. To sum up, ATR was effective against T2D in mouse, indicating its promising prospect in T2D treatment in the future.

## 4 DISCUSSION

T2D is a multilevel induced disease which was determined by disorder in several dimensions including metabolome and genome. The mechanism of T2D had been revealed, which was typically associated with the overeating of sugar, lipid.^16^ The constant and excessive stimulations of insulin secretion would end up with resistance to insulin which was the feature of T2D.^17^ Various methods, such as magnetic resonance spectroscopy and liquid and gas chromatography‐tandem mass spectrometry, had been conducted to explore effective treatments for T2D.

Metabolites were investigated in the treatments of disease for decades, which had proved the validity of metabolite profiling. Previous study identified serum and plasma amino acids as biomarkers of incident diabetes and insulin resistance, and elevated branched‐chain amino acids and glycine were supposed to be a robust biomarkers for diabetes.^18^ In a 3‐year cohort study, the onset mode of T2D was constructed by high‐resolution metabolomics, the cholesterol biosynthesis pathway was revealed as important factor in the progression of type 2 diabetes mellitus development.^19^ In addition, our study demonstrated that certain amino acids and energy dysregulations were altered in T2D patients, amino acids synthesis and degradation related metabolites such as malate were down‐regulated, and energy related metabolites such as ethanol were disorder.

Konstantopoulos et al^20^ proposed a novel method that reflected the insulin resistance level by gene expression signature scores, which indicated that the gene expression signature technology could be applied in patients’ characterization. Pilot study demonstrated that specific transcripts in blood might be associated with T2D, by detecting entire blood expression levels, T2D susceptibility as well as therapeutic response could be determined.^21^ Respectively, metabolomic and transcriptomic method had certain drawbacks, metabolomics could merely exert a phenotypical overview of disease which lacked for fundamental support and transcriptomic analysis was restricted by its specific research on genes, making the results susceptible to the altered progression of transcription, translation and modification. The integration of metabolomics and transcriptomics could make up for the deficiencies, by which we were capable of locating potential biomarkers and facilitating deeper insights into the cure of T2D. Calimlioglu et al^22^ investigated the biomarker signatures of tissue specific molecules, which revealed the connection between specific metabolites and signalling pathways. Previous studies discovered glycaemic deterioration as biomarkers in the progression of T2D. Based on data of DIRECT (Diabetes Research on Patient Stratification) Study, new therapeutic strategies were developed for the treatment of T2D.^23^

Contemporary studies focused on T2D through the integration of metabolomics and transcriptomics had certain limitations, since the thorough extraction of data from patients was time‐consuming and expensive, most studies were conducted in a small scale. Our study was structured on open databases which could be analysed and supported, thus gave solution to the deficiency in samples. Moreover, our study located specific genes or metabolites which were altered and might function in T2D progression, the discovery could provide certain previews for the discovery of biomarkers. Deficiencies were also obvious in our study. There existed differences between pathways from metabolomics and transcriptomics, the microarray assays were conducted in a small scale which might be interfered by random factors, and metabolomic analysis was supported by big data which was supposed to be more accurate. Moreover, metabolites were phenotypes of genes which directly related to disease mechanism, while the connection between gene alterations and diseases might be influenced by transcription and protein production. Further studies could emphasize on certain populations which lead to a more specific and accurate outcomes. With the application of emerging methods in this field, such as machine learning, more effective biomarkers as well as treatments would become reality.

The well‐established T2D animal models, db/db rice, represent primary hyperglycaemia similar to adult‐onset T2D due to a gene mutation influencing leptin receptors.^24^ Given this realization, we selected db/db to verify relative metabolism and transcription, as well as the effect of ATR on T2D. In the present study, ATR improved symptoms of T2D as a consequence of activated gene expression implicated to mitochondrial metabolism in T2D mice, suggesting genetic therapy may be a valuable idea. However, the kinetic of the ATR after administration was not determined in the present, which need to be further investigated to verify the treatment effect on ATR on T2D.

In summary, this study demonstrated results of metabolomic and transcriptomic analysis on patients with T2D and significant differences in db/db rice compared with control group. Treatment with ATR was able to reduce blood glucose possibly as a consequence of restored genes and pathways. Furthermore, these findings provided evidence for new therapy of type 2 diabetes.

## CONFLICTS OF INTEREST

The authors confirm that there are no conflicts of interest.

## AUTHOR CONTRIBUTIONS

HL, WC and HJ designed and conceptualized the study. XS, JK and MY analysed and interpreted the data. HP, HL and QL involved in statistical analysis. KY, ZC and HL drafted the manuscript. XS, HP and WC critically revised the manuscript. All authors have read and approved the final article.

## ETHICAL APPROVAL

All procedures performed in studies involving human participants and animals were in accordance with the ethical standards of Peking Union Medical College Hospital, Chinese Academy of Medical Sciences and Peking Union Medical College.

## CONSENT FOR PUBLICATION

Not applicable.

## Supporting information

Table S1

Table S2

Table S3

## Data Availability

The dataset supporting the conclusions of this article is available in the corresponding author repository.
