# Supplementary material for: CMap analysis identifies Atractyloside as a potential drug candidate for type 2 diabetes based on integration of metabolomics and transcriptomics
Source: J Cell Mol Med. 2020 May 29;24(13):7417–26. doi: 10.1111/jcmm.15357 (PMC7339182; doi:10.1111/jcmm.15357)
Supplement: Supplementary file 1 — Table S1 [file JCMM-24-7417-s001.docx]

**Supplementary table 1.** Analysis of metabolites

| metabolites | P.Value | adj.P.Val | logFC | t |
| --- | --- | --- | --- | --- |
| N-acetylglutamate | 0.00000 | 0.00000 | -0.20777 | -7.69033 |
| α-ketoisovaleric acid | 0.00000 | 0.00000 | 0.11647 | 6.60011 |
| ethanol | 0.00000 | 0.00000 | 0.11647 | 6.60011 |
| methyl guanidine | 0.00000 | 0.00003 | -0.09046 | -5.12289 |
| N-methylnicotinamide | 0.00001 | 0.00006 | -0.37614 | -4.86378 |
| hippurate | 0.00001 | 0.00010 | -1.30459 | -4.69244 |
| histidine | 0.00005 | 0.00040 | -0.14393 | -4.28369 |
| pyrimidine | 0.00014 | 0.00105 | 0.02845 | 3.98276 |
| malate | 0.00066 | 0.00353 | -1.35478 | -3.53575 |
| succinate | 0.00066 | 0.00353 | -0.05478 | -3.53575 |
| ureidopropionate | 0.00066 | 0.00353 | -0.05478 | -3.53575 |
| 2-hydroxybutyric acid | 0.00101 | 0.00498 | 0.19445 | 3.40349 |
| indoxyl sulphate | 0.00255 | 0.01119 | 0.29987 | 3.10892 |
| glycolate | 0.00304 | 0.01119 | -0.55615 | -3.05097 |
| indoxyl sulfate | 0.00320 | 0.01119 | 0.64323 | 3.03362 |
| fumarate | 0.00329 | 0.01119 | -0.00553 | -3.02437 |
| phenylalanine | 0.00340 | 0.01119 | 0.23150 | 3.01340 |
| 4-aminohippurate | 0.00360 | 0.01119 | 0.03362 | 2.99357 |
| desaminotyrosine | 0.00360 | 0.01119 | 0.03362 | 2.99357 |
| N-butyrate | 0.01097 | 0.03236 | 0.53508 | 2.60023 |
| isoleucine | 0.01473 | 0.03904 | 0.06270 | 2.48941 |
| acetoacetate | 0.01522 | 0.03904 | 0.14592 | 2.47683 |
| isocaproate | 0.01522 | 0.03904 | 0.14592 | 2.47683 |
| N-acetylaspartate | 0.01592 | 0.03914 | -0.24948 | -2.45957 |
| alanine | 0.01799 | 0.04245 | 0.13716 | 2.41230 |
| oxalacetate | 0.02118 | 0.04627 | 0.12977 | 2.34805 |
| pyruvate | 0.02118 | 0.04627 | 0.12977 | 2.34805 |
