# Supplementary material for: CMap analysis identifies Atractyloside as a potential drug candidate for type 2 diabetes based on integration of metabolomics and transcriptomics
Source: J Cell Mol Med. 2020 May 29;24(13):7417–26. doi: 10.1111/jcmm.15357 (PMC7339182; doi:10.1111/jcmm.15357)
Supplement: Supplementary file 2 — Table S2 [file JCMM-24-7417-s002.docx]

| **rank** | **cmap name** | **mean** | **n** | **enrichment** | **p** | **specificity** | **percent non-null** |
| --- | --- | --- | --- | --- | --- | --- | --- |
| 1 | atractyloside | -0.555 | 5 | -0.87 | 0.00008 | 0 | 100 |
| 2 | NS-398 | -0.578 | 3 | -0.918 | 0.00094 | 0 | 100 |
| 3 | ebselen | 0.537 | 3 | 0.905 | 0.00174 | 0 | 100 |
| 4 | thioridazine | 0.183 | 20 | 0.395 | 0.00246 | 0.5388 | 55 |
| 5 | epiandrosterone | 0.497 | 4 | 0.806 | 0.00265 | 0.0112 | 100 |
| 6 | suloctidil | 0.45 | 4 | 0.802 | 0.0029 | 0.0546 | 100 |
| 7 | geldanamycin | -0.307 | 15 | -0.446 | 0.00342 | 0.1797 | 60 |
| 8 | irinotecan | 0.605 | 3 | 0.868 | 0.00425 | 0.1909 | 100 |
| 9 | iproniazid | -0.389 | 5 | -0.712 | 0.00425 | 0.0075 | 80 |
| 10 | oxolamine | -0.492 | 4 | -0.785 | 0.00432 | 0.0231 | 100 |
| 11 | nabumetone | 0.499 | 4 | 0.78 | 0.00442 | 0.0065 | 100 |
| 12 | fluspirilene | 0.432 | 4 | 0.778 | 0.0046 | 0.005 | 100 |
| 13 | etodolac | -0.283 | 5 | -0.708 | 0.00467 | 0 | 60 |
| 14 | melatonin | -0.399 | 4 | -0.777 | 0.00511 | 0.0192 | 75 |
| 15 | corticosterone | -0.357 | 4 | -0.774 | 0.00533 | 0 | 75 |
| 16 | fluorocurarine | 0.383 | 4 | 0.762 | 0.00605 | 0.0083 | 100 |
| 17 | azacyclonol | 0.355 | 5 | 0.697 | 0.00615 | 0.0381 | 60 |
| 18 | spiramycin | -0.369 | 6 | -0.64 | 0.00634 | 0 | 66 |
| 19 | CP-944629 | 0.476 | 4 | 0.756 | 0.00682 | 0 | 75 |
| 20 | naltrexone | -0.44 | 5 | -0.684 | 0.00723 | 0.0393 | 80 |

**Supplementary Table 2. The ranking of CMap analysis**
