# Supplementary material for: CMap analysis identifies Atractyloside as a potential drug candidate for type 2 diabetes based on integration of metabolomics and transcriptomics
Source: J Cell Mol Med. 2020 May 29;24(13):7417–26. doi: 10.1111/jcmm.15357 (PMC7339182; doi:10.1111/jcmm.15357)
Supplement: Supplementary file 3 — Table S3 [file JCMM-24-7417-s003.docx]

**Supplementary Table 3. The effect of ATR on food intake of mice (g/day)**

|  | C57BL/6J | db/db | db/db+ATR |
| --- | --- | --- | --- |
| 0w | 4.13±0.15 | 8.21±0.23^**^ | 7.51±1.03 |
| 2w | 3.97±0.13 | 8.01±0.72^**^ | 7.38±0.74 |
| 4w | 4.21±0.12 | 7.93±0.45^**^ | 7.03±0.36 |
| 6w | 4.01±0.13 | 7.6±0.31^**^ | 6.51±0.24^#^ |
| 8w | 4.42±0.11 | 7.31±0.27^**^ | 6.33±0.22^#^ |
| 10w | 4.35±0.14 | 7.65±0.54^**^ | 6.22±0.27 |

The data were expressed as means ± S.E.M. (n = 5). ^**^ *P* < 0.01 *versus* C57BL/6J mice; ^#^ *P* < 0.05 *versus* db/db mice.
